# Real-world safety and effectiveness of nivolumab for advanced renal cell carcinoma in Japan: a post-marketing surveillance

**DOI:** 10.1007/s10147-022-02155-3

**Published:** 2022-04-20

**Authors:** Hirotsugu Uemura, Yoshihiko Tomita, Norio Nonomura, Kenji Yoshizaki, Takafumi Nakao, Nobuo Shinohara

**Affiliations:** 1 Department of Urology, Kindai University Faculty of Medicine, 377-2 Ohnohigashi, Osakasayama City, Osaka 589-8511 Japan; 2 Department of Urology, Department of Molecular Oncology, Niigata University Graduate School of Medical and Dental Sciences, 757 Ichibancho, Asahimachi-dori, Chuo Ward, Niigata City, Niigata 951-8510 Japan; 3 Department of Urology, Osaka University Graduate School of Medicine, 2-2 Yamadaoka, Suita, Osaka 565-0871 Japan; 4 Safety Management Pharmacovigilance Division, Ono Pharmaceutical Co., Ltd., 1-5, Dosho-machi 2-chome, Chuo-ku, Osaka, 541-8526 Japan; 5 Department of Renal and Genitourinary Surgery, Hokkaido University Graduate School of Medicine, Kita 15, Nishi 7, Kita-ku, Sapporo, 060-8638 Japan

**Keywords:** Effectiveness, Japan, Nivolumab, Post-marketing surveillance, Safety, Un-resectable or metastatic renal cell carcinoma

## Abstract

**Background:**

This all-case post-marketing surveillance (PMS) evaluated the real-world safety and effectiveness of nivolumab monotherapy in Japanese patients with un-resectable or metastatic renal cell carcinoma (RCC).

**Methods:**

This multicenter, open-label, non-interventional, observational PMS study (registered from August 2016 to January 2017) was conducted in patients who were newly initiated on nivolumab monotherapy. Assessments included treatment-related adverse events (TRAEs) of special interest, patient characteristics affecting safety, and effectiveness over 12 months.

**Results:**

Overall, 580 patients were enrolled; 555 and 554 patients comprised the safety and effectiveness analysis sets, respectively. The median (range) age of the population was 66 (14–90) years. Nivolumab was initiated as 1st-, 2nd-, and ≥ 3rd-line treatment in 0.2%, 42.0%, and 57.8% of patients, respectively. TRAEs were reported in 275 (49.5%) patients. The most common TRAEs of special interest included thyroid dysfunction (9.5%), hepatic dysfunction (8.6%), and interstitial lung disease (6.7%). The incidence of TRAEs was significantly higher in elderly patients (≥ 65 vs < 65 years; ≥ 75 vs < 75 years); patients with lower C-reactive protein levels (< 5 vs ≥ 5 mg/dL); and patients with vs without a past medical history, including hepatic, thyroid, and autoimmune diseases. The 6- and 12-month survival rates were 71.8% and 57.9%, respectively.

**Conclusion:**

The safety profile of nivolumab monotherapy in Japanese patients with advanced RCC was similar to that in the phase 3 CheckMate 025 trial. No new safety signals were observed in this study.

**Supplementary Information:**

The online version contains supplementary material available at 10.1007/s10147-022-02155-3.

## Introduction

Nivolumab is the world’s first human anti–programmed cell death protein 1 monoclonal antibody that was approved in Japan in 2014 for patients with malignant melanoma and in 2016 for patients with renal cell carcinoma. Currently, nivolumab is approved for numerous cancers in more than 65 countries worldwide.

A multinational phase 3 study (CheckMate 025 trial) conducted in patients with un-resectable or metastatic renal cell carcinoma (RCC) in Japan and other countries demonstrated the superior efficacy of nivolumab over everolimus. Median overall survival (OS) as the primary endpoint was 25.0 months with nivolumab and 19.6 months with everolimus. Fewer grade 3 or 4 treatment-related adverse events (TRAEs) were reported with nivolumab than with everolimus [1]. A subsequent subgroup analysis showed that in Japanese patients from the CheckMate 025 trial, the efficacy and safety of nivolumab versus everolimus were consistent with that reported in the global population [2]. Consequently, in August 2016, the manufacturing and marketing approval for nivolumab was updated to include the indications of un-resectable or metastatic RCC in Japan.

However, since the number of patients enrolled in the clinical trial was limited in Japan (37 patients with nivolumab) [2], an all-case post-marketing surveillance (PMS) study was mandated as a condition for approval. This is the first report of an all-case PMS for nivolumab in RCC conducted to evaluate the real-world safety, including TRAEs of special interest and risk factors of patient characteristics affecting safety, and effectiveness of nivolumab monotherapy in patients with un-resectable or metastatic RCC.

## Patients and methods

### Study design

This PMS was a multicenter, open-label, non-interventional, observational study conducted at 237 centers across Japan using a central registration system. Each participating center signed a contract with the sponsor to undertake this surveillance. The study complies with the ministerial ordinance of Good Post-Marketing Study Practice in Japan [3]. Written informed consent of patients and Institutional Review Board approval were waived as these were not required for the PMS study. Japanese patients with un-resectable or metastatic RCC who were to be treated with nivolumab between August 26, 2016, and January 31, 2017, were registered and observed for 12 months after initiating treatment with nivolumab. Patients who discontinued treatment before completion of the 12-month observation period were monitored up to the end of the observation period as frequently as possible.

### Patients

All patients with un-resectable or metastatic RCC who were newly initiated on nivolumab monotherapy for the prescribed indication (per prescribing information) were registered for this PMS study. Patients who had not been treated with systemic therapy before initiating nivolumab were excluded from the effectiveness analysis set.

### Assessments

Adverse events (AEs) were recorded 12 months after the start of treatment, and their relation to nivolumab was judged by each attending physician. Incidence of TRAEs in patients with RCC, particularly that of events described as important identified risks in the drug risk management plan, was captured by this PMS study. We used the Japanese version of the Medical Dictionary for Regulatory Activities version 22.1 and the National Cancer Institute Common Terminology Criteria for Adverse Events version 4.0 for classifying and grading each TRAE, respectively. Effectiveness was evaluated as the OS rate at 6 and 12 months after the first nivolumab dose.

### Statistical analysis

The incidence of TRAEs was characterized by patient background factors using the Fisher’s exact test, Wilcoxon rank-sum test, and chi-square test. Multivariate regression analyses evaluated the association of the occurrence of hepatic dysfunction, thyroid dysfunction, and interstitial lung disease (ILD) with various risk factors (multiple independent variables) using the Fine and Gray model [4].

For hepatic dysfunction, thyroid dysfunction, and ILD, we used the sub-distribution hazards model by Fine and Gray, in which the onset of AEs was defined as events and any death before the onset of adverse effects as competing risks. Cessation/termination was defined as discontinuation or the end of observation period other than the occurrence of the relevant adverse effect or death. The starting point of the survival period was defined as the initial date of nivolumab use. Statistical analyses of risk factors associated with AEs are presented in Online Resource 1. Survival rate could not be calculated by the Kaplan–Meier method, since data on the exact date of patients' death were not collected.

## Results

### Patients

**Fig. 1:**
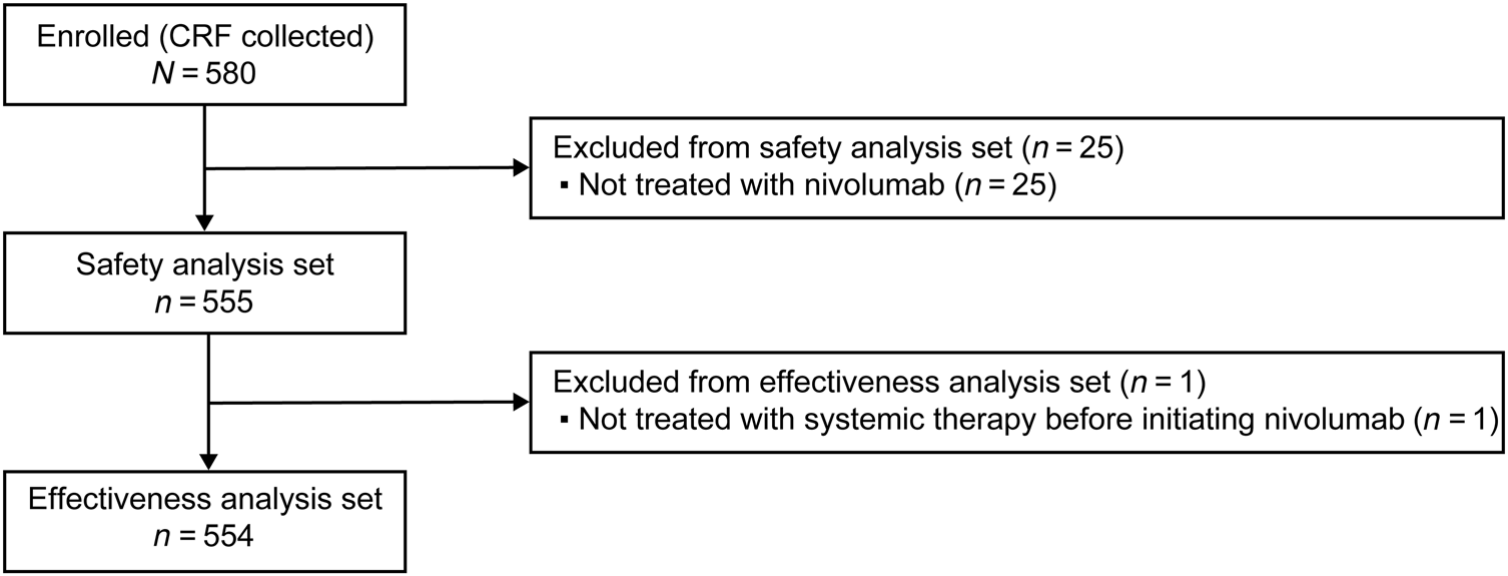
Patient disposition. *CRF* case report form

In total, 580 patients were enrolled between August 26, 2016, and January 31, 2017, from 237 facilities. After excluding patients not treated with nivolumab, 555 patients were included in the safety analysis set. The effectiveness analysis set comprised 554 patients after excluding one patient who was not treated with systemic therapy before initiating nivolumab (Fig. 1).

In the safety analysis set, median (range) age was 66 (14–90) years, with 14.8% of patients being ≥ 75 years; 23.1% of patients had a Karnofsky performance status (KPS) score of ≤ 70. The most common histological type of RCC was reported to be clear cell in 84.5% of patients, followed by papillary in 6.5% of patients. On the other hand, in the phase 3 CheckMate 025 trial, the median (range) age was 62 (23–88) years, with 8.3% of patients being ≥ 75 years; 5.9% of patients had a KPS score of ≤ 70, and all patients had clear cell RCC [1].

Nivolumab was initiated as 1st-, 2nd-, and ≥ 3rd-line treatment in 0.2%, 42.0%, and 57.8% of patients, respectively. A total of 378 (68.1%) patients had a “past medical history,” including 7.9% with liver and 14.1% with kidney disorders (Table 1).

**Table 1:** Patient demographics and baseline characteristics (safety analysis set)

Patient characteristics	*n* (%)
Overall, *N*	555 (100.0)
Sex	
Male	432 (77.84)
Female	123 (22.16)
Age (years)	
< 15	1 (0.18)
15 to < 65	236 (42.52)
65 to < 75	236 (42.52)
≥ 75	82 (14.77)
Karnofsky performance status	
100	148 (26.67)
90	185 (33.33)
80	94 (16.94)
70	57 (10.27)
60	30 (5.41)
50	26 (4.68)
40	7 (1.26)
30	4 (0.72)
20	1 (0.18)
10	3 (0.54)
Histological type of renal cell carcinoma^a^	
Clear cell	469 (84.50)
Papillary	36 (6.49)
Carcinoma of the collecting ducts of Bellini	3 (0.54)
Xp11.2 translocation carcinomas	3 (0.54)
Mucinous tubular and spindle cell carcinoma	5 (0.90)
Multi-locular clear cell	1 (0.18)
Chromo-phobe	5 (0.90)
Unclassified	9 (1.62)
Others	38 (6.85)
Past medical history	
No	175 (31.53)
Yes	378 (68.11)
Unknown	2 (0.36)
Past medical history: liver	
No	510 (91.89)
Yes	44 (7.93)
Unknown	1 (0.18)
Past medical history: kidney	
No	477 (85.95)
Yes	78 (14.05)
Treatment line	
1	1 (0.18)
2	233 (42.00)
≥ 3	321 (57.84)

^a^Patients with multiple histological types were counted separately; hence, the total of each category exceeds 555 patients

### Treatments

The mean ± standard deviation (median; minimum–maximum) number of nivolumab doses was 12.3 ± 9.3 (10; 1–31) in the safety analysis set. Among the < 75 years and ≥ 75 years age groups, 57.5% and 51.2% of patients received ≤ 12 mean number of nivolumab doses, respectively. In the C-reactive protein (CRP) < 5 mg/dL and CRP ≥ 5 mg/dL groups as well, 53.0% and 65.0% of patients received ≤ 12 mean number of nivolumab doses, respectively. A total of 155 (27.9%) patients continued treatment with nivolumab throughout the 12-month observation period, while 400 (72.1%) patients discontinued treatment. The reasons for nivolumab discontinuation, including those that were overlapping, were primary disease progression (including death; 41.3%), lack of effectiveness (19.6%), development of AEs (17.8%), hospital transfer (3.6%), confirmed effectiveness (1.4%), and others (1.3%).

### Safety

TRAEs (all grades) were reported in 275 (49.5%) patients. TRAEs with a frequency of ≥ 2% are presented in Online Resource 2. The most common TRAEs of special interest included thyroid dysfunction (9.5%), hepatic dysfunction (8.6%), and ILD (6.7%). The most common grade ≥ 3 TRAEs of special interest included hepatic dysfunction (3.1%), ILD (3.1%), and colitis/severe diarrhea (2.0%; Table 2). Deaths due to TRAEs were reported in 14 (2.5%) patients. Of these, 7 (1.3%) patients died due to grade 5 TRAEs of special interest, such as cardiac disorders (0.5%), ILD (0.4%), hepatic dysfunction (0.2%), and infusion reaction (0.2%).

**Table 2:** Incidence of TRAEs of special interest by grade (safety analysis set)

TRAEs of special interest	Grade 1	Grade 2	Grade 3	Grade 4	Grade 5	Unknown	Total
	*n* (%)	*n* (%)	*n* (%)	*n* (%)	*n* (%)	*n* (%)	*n* (%)
Overall	60 (10.81)	67 (12.07)	54 (9.73)	11 (1.98)	7 (1.26)	6 (1.08)	205 (36.94)
Interstitial lung disease	7 (1.26)	11 (1.98)	10 (1.80)	5 (0.90)	2 (0.36)	2 (0.36)	37 (6.67)
Myasthenia gravis/myocarditis/ myositis/rhabdomyolysis	1 (0.18)	0 (0)	2 (0.36)	0 (0)	0 (0)	0 (0)	3 (0.54)
Colitis/severe diarrhea	8 (1.44)	8 (1.44)	11 (1.98)	0 (0)	0 (0)	2 (0.36)	29 (5.23)
Type 1 diabetes mellitus	0 (0)	0 (0)	1 (0.18)	1 (0.18)	0 (0)	0 (0)	2 (0.36)
Hepatic dysfunction	19 (3.42)	11 (1.98)	12 (2.16)	4 (0.72)	1 (0.18)	1 (0.18)	48 (8.65)
Thyroid dysfunction	18 (3.24)	31 (5.59)	2 (0.36)	0 (0)	0 (0)	2 (0.36)	53 (9.55)
Renal disorder	8 (1.44)	9 (1.62)	7 (1.26)	1 (0.18)	0 (0)	0 (0)	25 (4.50)
Adrenal dysfunction	2 (0.36)	3 (0.54)	7 (1.26)	0 (0)	0 (0)	2 (0.36)	14 (2.52)
Severe skin disorders	0 (0)	1 (0.18)	3 (0.54)	0 (0)	0 (0)	1 (0.18)	5 (0.90)
Venous thromboembolism	1 (0.18)	0 (0)	0 (0)	0 (0)	0 (0)	0 (0)	1 (0.18)
Infusion reaction	19 (3.42)	9 (1.62)	0 (0)	0 (0)	1 (0.18)	0 (0)	29 (5.23)
Cardiac disorder	0 (0)	3 (0.54)	4 (0.72)	1 (0.18)	3 (0.54)	2 (0.36)	13 (2.34)

TRAEs are presented using the MedDRA/J version 22.1

Patients experiencing the same TRAEs multiple times were included in the highest-grade category among the events

*TRAE* treatment-related adverse event, *MedDRA/J* Japanese version of the Medical Dictionary for Regulatory Activities

Incidences of TRAE of special interest in this PMS study and the CheckMate 025 trial are shown in Online Resource 3. Among TRAEs of special interest, the incidence of all-grade ILD, adrenal disorder, and cardiac disorder in this PMS population (6.7%, 2.5%, and 2.3%, respectively) was higher to an extent (1.0% or more) than that in the overall population of the CheckMate 025 trial (4.9%, 1.5%, and 1.2%, respectively). Of the 37 patients who developed ILD in this PMS, the outcome was recovered/improved in 29, recovered with sequelae in 1, not recovered in 5, and death in 2. Of the 14 patients who developed adrenal disorders (adrenal insufficiency, *n* = 13; secondary adrenocortical insufficiency, *n* = 1) in this PMS, the outcome was recovered/improved in 11, not recovered in 1, and unknown in 2. Of the 13 patients who developed cardiac disorders in this PMS, the outcome was recovered/improved in 9, death in 3, and unknown in 1.

In addition, 13.5% (5/37) of the patients who developed ILD had a history of ILD. Of the 14 patients who developed adrenal disorders, none had a history of adrenal disorders or adrenal metastases. Of the patients who developed cardiac disorders, 23.1% (3/13) had a history of cardiac disease (congestive heart failure, *n* = 2; heart failure, *n* = 1).

### TRAEs categorized by patient background factors

The incidences of TRAEs in patients categorized by background factors are shown in Table 3. A significant difference (*p* < 0.05) was observed in the incidence rate of TRAEs among patients stratified by age (< 65 years [44.3%] vs ≥ 65 years [53.5%]; < 75 years [47.6%] vs ≥ 75 years [61.0%]); CRP levels (< 5 mg/dL [53.8%] vs ≥ 5 mg/dL [43.8%]); and past medical history (presence [54.5%] vs absence [39.4%]), including hepatic diseases (65.9% vs 48.0%), thyroid diseases (58.0% vs 46.8%), and autoimmune diseases (75.0% vs 48.8%; Table 3).

**Table 3:** Incidence of TRAEs by patient background factors (safety analysis set)

Patient background factors	*n* (%)	TRAE incidence				
		*n*	Incidence rate (%)	95% CI of the incidence rate^a^	*p* value	Test method
Safety analysis set	555 (100.00)	275	49.55	45.31–53.79		
Age (years)						
Mean ± SD	64.3 ± 11.0					
Median	66.00					
Min–max	14–90					
Age (years)						
< 65	237 (42.70)	105	44.30	37.88–50.88	*p* = 0.0330^b^	W
≥ 65	318 (57.30)	170	53.46	47.81–59.04		
Age (years)						
< 75	473 (85.23)	225	47.57	42.99–52.18	*p* = 0.0251^b^	W
≥ 75	82 (14.77)	50	60.98	49.57–71.56		
Past medical history						
No	175 (31.53)	69	39.43	32.14–47.08	*p* = 0.0010^b^	F
Yes	378 (68.11)	206	54.50	49.33–59.60		
Unknown	2 (0.36)	0	–	–		
Past medical history: liver						
No	510 (91.89)	245	48.04	43.63–52.47	*p* = 0.0274^b^	F
Yes	44 (7.93)	29	65.91	50.08–79.51		
Unknown	1 (0.18)	1	100.00	–		
Past medical history: thyroid						
No	417 (75.14)	195	46.76	41.89–51.68	*p* = 0.0241^b^	F
Yes	138 (24.86)	80	57.97	49.28–66.32		
Past medical history: autoimmune disease						
No	539 (97.12)	263	48.79	44.50–53.10	*p* = 0.0442^b^	F
Yes	16 (2.88)	12	75.00	47.62–92.73		
CRP (before using nivolumab; mg/dL)						
Number of patients	507					
Mean ± SD	3.636 ± 4.642					
Median	1.60					
Min–max	0.00–23.61					
CRP (before using nivolumab; mg/dL)						
< 5	370 (66.67)	199	53.78	48.56–58.95	*p* = 0.0460^b^	W
≥ 5	137 (24.68)	60	43.80	35.34–52.53		
Unknown	48 (8.65)	16	33.33	–		

*CI* confidence interval, *CRP* C-reactive protein, *F* Fisher’s exact test, *max* maximum, *min* minimum, *SD* standard deviation, *TRAE* treatment-related adverse event, *W* Wilcoxon rank-sum test

^a^95% CIs were calculated by Fisher’s exact test

^b^*p* < 0.05 was considered significant

Multivariate regression analyses showed that the risk of hepatic dysfunction was significantly higher among patients with versus without a past medical history of hepatic disease (Online Resource 4). Further, these analyses showed that the risk of thyroid dysfunction was significantly higher among patients with versus without a past medical history of thyroid disease and among those aged ≥ 75 years versus < 75 years (Online Resource 5). Similarly, multivariate analyses showed that the risk of ILD was significantly higher among patients with versus without a past medical history of ILD and autoimmune diseases (Online Resource 6).

### Effectiveness

In this PMS study, the 6- and 12-month survival rates (95% confidence interval) were 71.8% (67.9%–75.6%) and 57.9% (53.7%–62.1%), respectively (Table 4).

**Table 4:** Overall survival rate during the observation period (effectiveness analysis set)

Period	Number of survived patients/N	Survival rate (%)	95% CI of the survival rate^a^
6 months	398/554	71.84	67.90–75.55
12 months	321/554	57.94	53.71–62.09

*CI* confidence interval

^a^Calculated using the Fisher’s exact method

## Discussion

This large PMS study clarified the real-world safety and effectiveness of nivolumab in Japanese patients with advanced RCC with results similar to those observed in the phase 3 CheckMate 025 trial and another retrospective real-world study [1, 2, 5].

Among TRAEs of special interest, the incidence of all-grade ILD, adrenal disorder, and cardiac disorder in this PMS population was higher to an extent than that in the overall population of the CheckMate 025 trial. In contrast, the incidence of all-grade ILD in the Japanese population of the CheckMate 025 trial (8.1%) was comparable to that in this PMS population (6.7%). The prevalence of drug-induced pneumonia has been reported to be relatively high in the Japanese population [6], suggesting that the higher incidence of ILD in this PMS compared with that in the global population could be attributed to the influence of differences in patient background factors.

No patients who developed adrenal disorders had a history of adrenal disorders or adrenal metastases. The relationship between history of adrenal disorders and the occurrence of AEs has not been analyzed in this PMS study or in the phase 3 CheckMate 025 trial and the aforementioned other retrospective real-world study [1, 2, 5]. However, in most patients, the adrenal disorders recovered/improved during the study period, suggesting that these AEs could be managed appropriately according to existing AE management algorithms [7]. Nevertheless, since adrenal disorders are often exacerbated, subjective symptoms, such as malaise, consciousness disturbed, and nausea/vomiting, should be carefully monitored for early detection.

It is known that in general, cardiac disorders are more likely to occur with molecular targeted drugs. The incidence of cardiac disorders was 2.3% in this PMS study and 1.2% in the CheckMate 025 trial [1]. The eligibility criteria for the CheckMate 025 trial stipulated the inclusion of “Patients with one or two angiogenesis inhibitors (including, but not limited to, sunitinib, pazopanib, and axitinib) as treatment for advanced or metastatic RCC, but not more than three prior regimens, with or without prior treatment with cytokine therapy (e.g., interleukin-2 and interferon-alpha), vaccine therapy, or cytotoxic anticancer drugs.” As prior therapies in the CheckMate 025 trial, 72.0% of patients received one angiogenesis inhibitor and 28.0% of patients received two angiogenesis inhibitors [1]. On the other hand, in this PMS, where 57.8% of patients received nivolumab as ≥ 3rd-line treatment, prior therapies included mammalian target of rapamycin inhibitors or cytokine therapies in addition to angiogenesis inhibitors. Although direct comparison is difficult, it is likely that more patients in this PMS used more numbers of molecular targeted drugs compared with the patients in the CheckMate 025 trial [1]. In addition, 23.1% of patients who developed cardiac disorders had a history of cardiac disease. Based on the above, the higher incidence of cardiac disorders in this PMS may be attributed to differences in patient background factors, such as prior treatment, past medical history, and comorbidities.

We observed that the incidence of TRAEs was significantly lower in the younger age group (< 65 or < 75 years) versus the older age group (≥ 65 or ≥ 75 years). Cross-tabulation with the number of nivolumab doses showed that the younger age group received a lower number of doses (data not shown). Similarly, the incidence of TRAEs was lower in the CRP ≥ 5 mg/dL versus CRP < 5 mg/dL group, and cross-tabulation with the number of nivolumab doses showed that patients in the CRP ≥ 5 mg/dL group received a lower number of nivolumab doses (data not shown). In the younger group and the CRP ≥ 5 mg/dL group, many patients were considered to have died or stopped the therapy before the onset of adverse reactions, which may have affected the incidence of adverse reactions.

Furthermore, patients with a “past medical history” and/or “present comorbidities” related to an organ are likely to experience more TRAEs due to organ dysfunction. Multivariate analyses confirmed that patients with a past medical history of hepatic/thyroid dysfunction may experience significantly more TRAEs. Similarly, patients with a past medical history related to ILD may experience significantly more TRAEs.

Overall, no new safety signals were identified. Although some TRAEs occurred more frequently in this PMS study than in the CheckMate 025 trial, no specific concerns were raised.

The 6- and 12-month survival rates (71.8% and 57.9%) in this study were lower than those in the CheckMate 025 trial (89.2% and 76.0%), respectively. Although direct comparison may not be appropriate partly due to the different evaluation methods, these differences in the survival rates may be attributed to different patient characteristics, such as KPS score, histological types, and age, that are known to be related to poor prognosis; this PMS study included more patients with poor (≤ 70) KPS score (23.1% vs 5.9%), histological types other than clear cell type (15.5% vs none), and age ≥ 75 years (14.8% vs 8.3%) compared with the CheckMate 025 trial [1]. The effectiveness in a real-world setting may help us understand the net benefit of nivolumab for un-resectable or metastatic RCC patients. Effectiveness in this study was evaluated by the 1-year OS rate since the follow-up period of the study was up to 1 year, which made it difficult to evaluate long-term effectiveness of the immuno-oncology therapy. In addition, neither overall response rate nor progression-free survival was evaluated in this study because mandating the computed tomography (CT) evaluation for Response Evaluation Criteria in Solid Tumors at a certain timing was difficult in PMS. Thus, it is not appropriate to conclude the real-world effectiveness of nivolumab solely by this PMS. Further analysis of real-world data with longer follow-up time is needed to evaluate the long-term effectiveness of nivolumab in future.

### Limitations

This was a PMS study, and treatment was based on the physician’s discretion. We did not conduct a central review of the TRAEs in case report forms. Thus, TRAE incidences may be underestimated or overestimated compared with those reported in clinical trials. In addition, in this PMS study, the acquisition of CT images was limited.

## Conclusion

The results of this PMS study showed that the safety of nivolumab monotherapy in Japanese patients with un-resectable or metastatic RCC was similar to that observed in the phase 3 CheckMate 025 trial and another retrospective real-world study. No new safety concerns were identified over the 12-month observation period in patients with RCC. These results support nivolumab as a treatment option for patients with advanced RCC in the real world.

## Supplementary Information

Below is the link to the electronic supplementary material.

Supplementary file1 (DOCX 44 KB)
