# Supplementary material for: Real-world safety and effectiveness of nivolumab for advanced renal cell carcinoma in Japan: a post-marketing surveillance
Source: Int J Clin Oncol. 2022 Apr 20;27(6):1061–7. doi: 10.1007/s10147-022-02155-3 (PMC9119881; doi:10.1007/s10147-022-02155-3)
Supplement: Supplementary file 1 — Supplementary file1 (DOCX 44 KB) [file 10147_2022_2155_MOESM1_ESM.docx]

**Online Resource**

**Online Resource 1** Statistical analysis of risk factors associated with adverse events

| The following procedures were used to explore the risk factors related to hepatic dysfunction: (1) Factor(s) was/were excluded from candidate risk factors when the following conditions were met: i) missing values of 10% or more and ii) Spearman’s rank correlation coefficient of 0.95 or higher and (2) The following methods were used to search for risk factors for the onset/development of hepatic dysfunction: A multivariate analysis was conducted using the subdistribution hazards model by Fine and Gray, in which the onset of adverse effects was defined as events and any death before the onset of adverse effects as competing risks. “History of disease (liver)” was included in the model as a risk factor because of its relatively high hazard ratio and clinical significance. In addition, we analyzed with a total of two variables, in which each item listed in “candidate risk factors for the development of hepatic dysfunction” was added as an explanatory variable to “past medical history (liver).” The determination of risk factors per the results of the multivariate analysis described above was based on the criterion that the 95% confidence interval of the hazard ratio does not exceed 1.  The risk factor for the development of hepatic dysfunction was the presence of past medical history (liver); candidate risk factors for the development of hepatic dysfunction were sex, age (< 75 and ≥ 75 years), Karnofsky performance status (KPS) score (100–80 and 70–10), history of autoimmune disease, presence of metastasis, treatment line (2nd and ≥ 3rd), and C-reactive protein (CRP) levels (before use of the drug; < 5 and ≥ 5 mg/dL).  To search for risk factors related to the onset/development of thyroid dysfunction, the same analysis as in (1) above was conducted. There were no prespecified risk factors for thyroid dysfunction. Therefore, all candidate risk factors were included in the subdistribution hazards model by Fine and Gray, and hazard ratios and their 95% confidence intervals were calculated for each factor.  Candidate risk factors for the development of thyroid dysfunction were sex, age (< 75 and ≥ 75 years), KPS score (100–80 and 70–10), history of each disease (thyroid, autoimmune), and treatment line (2nd and ≥ 3rd).  To search for risk factors related to the onset/development of ILD the same analyses as in (1) and (2) above were conducted.  The risk factor for the onset/development of ILD was the presence of ILD; candidate risk factors for the onset/development of ILD were sex, age (< 75 and ≥ 75 years), KPS score (100–80 and 70–10), presence of each past medical history (lung infection/autoimmune disease), presence of abnormal findings on chest imaging (computed tomography), presence of treatment (radiation therapy) for RCC in the past, treatment line (2nd and ≥ 3rd), prior use of molecular targeted drugs, prior use of mammalian target of rapamycin inhibitors, and CRP levels (before use of the drug; < 5 and ≥ 5 mg/dL). |
| --- |

**Online Resource 2** List of TRAE incidences (≥ 2%; safety analysis set)

|  |  | **Status up to approval (CheckMate 025)^a^** | **Status during PMS** |
| --- | --- | --- | --- |
| **SOC** | **PT** | ***n* (%)** | ***n* (%)** |
| **Safety analysis set** |  | 406 (100.00) | 555 (100.00) |
| **Patients with TRAEs** |  | 319 (78.57) | 275 (49.55) |
| **Blood and lymphatic system disorders** | Anemia | 32 (7.88) | 5 (0.90) |
| **Endocrine disorders** | Adrenal insufficiency | 6 (1.48) | 13 (2.34) |
|  | Hypothyroidism | 24 (5.91) | 41 (7.39) |
| **Metabolism and nutrition disorders** | Hyperglycemia | 9 (2.22) | 0 (0) |
|  | Decreased appetite | 48 (11.82) | 8 (1.44) |
| **Nervous system disorders** | Dizziness | 11 (2.71) | 1 (0.18) |
|  | Headache | 24 (5.91) | 2 (0.36) |
| **Respiratory, thoracic, and mediastinal disorders** | Cough | 36 (8.87) | 4 (0.72) |
|  | Dyspnea | 30 (7.39) | 5 (0.90) |
|  | Interstitial lung disease | 2 (0.49) | 32 (5.77) |
|  | Pneumonitis | 16 (3.94) | 0 (0) |
| **Gastrointestinal disorders** | Constipation | 24 (5.91) | 4 (0.72) |
|  | Diarrhea | 50 (12.32) | 24 (4.32) |
|  | Dry mouth | 16 (3.94) | 2 (0.36) |
|  | Nausea | 57 (14.04) | 6 (1.08) |
|  | Vomiting | 24 (5.91) | 7 (1.26) |
| **Hepatobiliary disorders** | Hepatic function abnormal | 1 (0.25) | 22 (3.96) |
| **Skin and subcutaneous tissue disorders** | Dermatitis acneiform | 12 (2.96) | 1 (0.18) |
|  | Dry skin | 26 (6.40) | 5 (0.90) |
|  | Erythema | 11 (2.71) | 3 (0.54) |
|  | Pruritus | 59 (14.53) | 10 (1.80) |
|  | Rash | 45 (11.08) | 16 (2.88) |
|  | Rash maculopapular | 16 (3.94) | 1 (0.18) |
| **Musculoskeletal and connective tissue disorders** | Arthralgia | 27 (6.65) | 3 (0.54) |
|  | Myalgia | 23 (5.67) | 3 (0.54) |
| **Renal and urinary disorders** | Renal impairment | 1 (0.25) | 16 (2.88) |
| **General disorders and administration-site conditions** | Asthenia | 18 (4.43) | 0 (0) |
|  | Chills | 20 (4.93) | 0 (0) |
|  | Fatigue | 134 (33.00) | 2 (0.36) |
|  | Malaise | 6 (1.48) | 19 (3.42) |
|  | Mucosal inflammation | 11 (2.71) | 0 (0) |
|  | Edema peripheral | 17 (4.19) | 2 (0.36) |
|  | Pyrexia | 34 (8.37) | 15 (2.70) |
| **Investigations** | Alanine aminotransferase increased | 16 (3.94) | 10 (1.80) |
|  | Aspartate aminotransferase increased | 19 (4.68) | 9 (1.62) |
|  | Blood creatinine increased | 27 (6.65) | 6 (1.08) |
|  | Lymphocyte count decreased | 9 (2.22) | 0 (0) |
|  | Weight decreased | 19 (4.68) | 0 (0) |
|  | Blood alkaline phosphatase increased | 17 (4.19) | 8 (1.44) |
| **Injury, poisoning, and procedural complications** | Infusion-related reaction | 13 (3.20) | 8 (1.44) |

^a^Four patients were excluded as they did not receive nivolumab.

*PMS* postmarketing surveillance; *PT* preferred term; *SOC* System Organ Class; *TRAE* treatment-related adverse event.

**Online Resource 3** List of TRAE incidences of special interest

| TRAEs of special interest | Current PMS study  (*N* = 555) | | CheckMate 025 (global)^a^  (*N* = 406) | | CheckMate 025 (Japanese)^a^  (*N* = 37) | |
| --- | --- | --- | --- | --- | --- | --- |
|  | **Total**  ***n* (%)** | **Grade ≥ 3**  ***n* (%)** | **Total**  ***n* (%)** | **Grade ≥ 3**  ***n* (%)** | **Total**  ***n* (%)** | **Grade ≥ 3**  ***n* (%)** |
| Interstitial lung disease | 37 (6.67) | 17 (3.06) | 20 (4.93) | 7 (1.72) | 3 (8.11) | 0 |
| Myasthenia gravis/myocarditis/ myositis/rhabdomyolysis | 3 (0.54) | 2 (0.36) | 1 (0.25) | 0 | 0 | 0 |
| Colitis/severe diarrhea | 29 (5.23) | 11 (1.98) | 51 (12.56) | 8 (1.97) | 5 (13.51) | 1 (2.70) |
| Type I diabetes mellitus | 2 (0.36) | 2 (0.36) | 1 (0.25) | 1 (0.25) | 0 | 0 |
| Hepatic dysfunction | 48 (8.65) | 17 (3.06) | 48 (11.82) | 11 (2.71) | 9 (24.32) | 2 (5.41) |
| Thyroid dysfunction | 53 (9.55) | 2 (0.36) | 35 (8.62) | 1 (0.25) | 2 (5.41) | 0 |
| Renal disorder | 25 (4.50) | 8 (1.44) | 31 (7.64) | 5 (1.23) | 1 (2.70) | 0 |
| Adrenal dysfunction | 14 (2.52) | 7 (1.26) | 6 (1.48) | 2 (0.49) | 0 | 0 |
| Severe skin disorders | 5 (0.90) | 3 (0.54) | 4 (0.99) | 0 | 3 (8.11) | 0 |
| Venous thromboembolism | 1 (0.18) | 0 | 0 | 0 | 0 | 0 |
| Infusion reaction | 29 (5.23) | 1 (0.18) | 144 (35.47) | 6 (1.48) | 10 (27.03) | 0 |
| Cardiac disorder | 13 (2.34) | 8 (1.44) | 5 (1.23) | 1 (0.25) | 0 | 0 |

If the same TRAE of special interest occurred more than once in the same patient, it was counted using the date of initial onset.

^a^The period from the date of initial dose of nivolumab to 30 days after the last dose of the drug was included in the analysis.

*TRAE* treatment-related adverse event.

**Online Resource 4** Multivariate regression analysis of occurrence of hepatic dysfunction using the Fine and Gray model

| Candidate risk factors | Explanatory variable | Comparison | HR | 95% CI of HR |
| --- | --- | --- | --- | --- |
|  | Medical history: liver | Present vs absent | 3.47 | 1.75–6.89 |
| → | Sex | Male vs female | 0.68 | 0.36–1.29 |
|  | Medical history: liver | Present vs absent | 3.34 | 1.69–6.61 |
| → | Age (years) | ≥ 75 years vs < 75 years | 1.58 | 0.80–3.15 |
|  | Medical history: liver | Present vs absent | 3.24 | 1.61–6.54 |
| → | KPS category | 100–80 vs 70–10 | 1.63 | 0.73–3.65 |
|  | Medical history: liver | Present vs absent | 3.40 | 1.70–6.82 |
| → | Medical history: autoimmune disease | Present vs absent | 0.58 | 0.07–4.51 |
|  | Medical history: liver | Present vs absent | 3.38 | 1.70–6.73 |
| → | Metastasis | Present vs absent | 0.31 | 0.05–1.92 |
|  | Medical history: liver | Present vs absent | 3.25 | 1.65–6.40 |
| → | Treatment line | 2nd vs ≥ 3rd | 0.84 | 0.46–1.51 |
|  | Medical history: liver | Present vs absent | 3.81 | 1.91–7.62 |
| → | CRP^a^ (mg/dL) | ≥ 5 vs < 5 | 0.83 | 0.41–1.68 |

^a^Before use of nivolumab.

*CI* confidence interval; *CRP* C-reactive protein; *HR* hazard ratio; *KPS* Karnofsky performance status.

**Online Resource 5** Multivariate regression analysis of occurrence of thyroid dysfunction using the Fine and Gray model

| Candidate risk factors | Explanatory variable | Comparison | HR | 95% CI of HR |
| --- | --- | --- | --- | --- |
| → | Sex | Male vs female | 1.01 | 0.52–1.97 |
| → | Age (years) | ≥ 75 years vs < 75 years | 2.11 | 1.12–3.95 |
| → | KPS category | 100–80 vs 70–10 | 0.73 | 0.40–1.32 |
| → | Past medical history: thyroid | Present vs absent | 1.95 | 1.11–3.42 |
| → | Past medical history: autoimmune disease | Present vs absent | 1.42 | 0.31–6.43 |
| → | Treatment line | 2nd vs ≥ 3rd | 1.53 | 0.90–2.63 |

*CI* confidence interval; *HR* hazard ratio; *KPS* Karnofsky performance status.

**Online Resource 6** Multivariate regression analysis of occurrence of ILD using the Fine and Gray model

| Candidate risk factors | Explanatory variable | Comparison | HR | 95% CI of HR |
| --- | --- | --- | --- | --- |
|  | Past medical history: ILD | Present vs absent | 4.71 | 1.81–12.23 |
| → | Sex | Male vs female | 1.23 | 0.55–2.75 |
|  | Past medical history: ILD | Present vs absent | 4.69 | 1.82–12.11 |
| → | Age (years) | ≥ 75 years vs < 75 years | 0.93 | 0.37–2.38 |
|  | Past medical history: ILD | Present vs absent | 4.38 | 1.71–11.26 |
| → | KPS category | 100–80 vs 70–10 | 2.35 | 0.84–6.61 |
|  | Past medical history: ILD | Present vs absent | 4.79 | 1.85–12.39 |
| → | Past medical history: lung infection | Present vs absent | 1.86 | 0.24–14.66 |
|  | Past medical history: ILD | Present vs absent | 5.06 | 1.95–13.17 |
| → | Past medical history: autoimmune disease | Present vs absent | 3.47 | 1.11–10.92 |
|  | Past medical history: ILD | Present vs absent | 4.92 | 1.91–12.71 |
| → | Abnormal findings on chest imaging (CT) | Present vs absent | 1.01 | 0.51–2.00 |
|  | Past medical history: ILD | Present vs absent | 4.83 | 1.85–12.63 |
| → | Past treatment for RCC (radiation therapy) | Present vs absent | 0.88 | 0.42–1.84 |
|  | Past medical history: ILD | Present vs absent | 4.54 | 1.78–11.55 |
| → | Treatment line | 2nd vs ≥ 3rd | 0.80 | 0.41–1.57 |
|  | Past medical history: ILD | Present vs absent | 4.84 | 1.87–12.52 |
| → | Past use of molecular targeted drugs | Present vs absent | 0.24 | 0.03–1.81 |
|  | Past medical history: ILD | Present vs absent | 4.68 | 1.81–12.13 |
| → | Past use of mTOR inhibitors | Present vs absent | 1.07 | 0.52–2.21 |
|  | Past medical history: ILD | Present vs absent | 5.54 | 2.12–14.47 |
| → | CRP^a^ (mg/dL) | ≥ 5 vs < 5 | 0.89 | 0.41–1.94 |

^a^Evaluated before using nivolumab.

*CI* confidence interval; *CRP* C-reactive protein; *CT* computed tomography; *HR* hazard ratio; *ILD* interstitial lung disease; *KPS* Karnofsky performance status; *mTOR* mammalian target of rapamycin; *RCC* renal cell carcinoma.
